# Use of Procalcitonin during the First Wave of COVID-19 in the Acute NHS Hospitals: A Retrospective Observational Study

**DOI:** 10.3390/antibiotics10050516

**Published:** 2021-05-01

**Authors:** Neil Powell, Philip Howard, Martin J. Llewelyn, Tamas Szakmany, Mahableswhar Albur, Stuart E Bond, Joanne Euden, Lucy Brookes-Howell, Paul Dark, Thomas P Hellyer, Susan Hopkins, Iain J McCullagh, Margaret Ogden, Philip Pallmann, Helena Parsons, David G Partridge, Dominick E. Shaw, Bethany Shinkins, Stacy Todd, Emma Thomas-Jones, Robert West, Enitan D Carrol, Jonathan A. T. Sandoe

**Affiliations:** 1 Pharmacy Department, Royal Cornwall Hospital Trust, Truro TR1 3LJ, UK; 2 School of Healthcare, Faculty of Medicine and Health, University of Leeds, Leeds LS2 9JT, UK; philip.howard2@nhs.net; 3 Department of Medicines Management and Pharmacy, Leeds Teaching Hospitals, Leeds General Infirmary, Leeds LS1 3EX, UK; 4 Brighton and Sussex Medical School, University of Sussex, Brighton BN1 9PS, UK; m.j.llewelyn@bsms.ac.uk; 5 Grange University Hospital, Aneurin Bevan University Health Board, Llanyravon, Cwmbran NP44 2XJ, UK; szakmanyt1@cardiff.ac.uk; 6 Department of Anaesthesia, Intensive Care and Pain Medicine, Division of Population Medicine, Cardiff University, Cardiff CF14 4XN, UK; 7 North Bristol NHS Trust, Bristol BS10 5NB, UK; Mahableshwar.Albur@nbt.nhs.uk; 8 Mid Yorkshire Hospitals NHS Trust, Wakefield WF1 4DG, UK; stuart.bond@nhs.net; 9 School of Applied Sciences, University of Huddersfield, Huddersfield HD13DH, UK; 10 Centre for Trials Research, Neuadd Meirionydd, Cardiff University, Heath Park, Cardiff CF14 4YS, UK; EudenJ@cardiff.ac.uk (J.E.); Brookes-HowellLC@cardiff.ac.uk (L.B.-H.); pallmannp@cardiff.ac.uk (P.P.); Thomas-JonesE@cardiff.ac.uk (E.T.-J.); 11 Manchester NIHR Biomedical Research Centre, University of Manchester, Manchester M13 9PL, UK; paul.m.dark@manchester.ac.uk; 12 Translational and Clinical Research Institute, Newcastle University, Newcastle Upon Tyne NE1 7RU, UK; Thomas.Hellyer@newcastle.ac.uk; 13 Public Health England, London SE1 8UG, UK; susan.hopkins@phe.gov.uk; 14 The Newcastle upon Tyne hospitals NHS Foundation Trust, Newcastle Upon Tyne NE7 7DN, UK; i.mccullagh@nhs.net; 15 Patient and Public Involvement Representative, NIHR, London SW1A 2NS, UK; margaretogden@hotmail.com; 16 Sheffield Teaching Hospitals NHS Foundation Trust, Sheffield S10 2JF, UK; helena.parsons@nhs.net (H.P.); dpartridge@nhs.net (D.GP.); 17 Division of Respiratory Medicine, University of Nottingham, Nottingham NG7 2RD, UK; dominic.shaw@nottingham.ac.uk; 18 Test Evaluation Group, Leeds Institute of Health Sciences, University of Leeds, Leeds LS2 9JT, UK; B.Shinkins@leeds.ac.uk; 19 Liverpool University Hospital NHS Foundation Trust, Liverpool L9 7AL, UK; Stcy.Todd@liverpoolft.nhs.uk; 20 Leeds Institute of Health Sciences, University of Leeds, Leeds LS2 9TJ, UK; R.M.West@leeds.ac.uk; 21 Department of Clinical Infection Microbiology and Immunology, Institute of Infection, Veterinary & Ecological Sciences, University of Liverpool, Liverpool L69 3BX, UK; Edcarrol@liverpool.ac.uk; 22 Healthcare Associated Infection Group, Leeds Institute of Medical Research, School of Medicine, University of Leeds, LS2 9JT, UK; J.Sandoe@leeds.ac.uk

**Keywords:** antibiotic, stewardship, COVID-19, procalcitonin

## Abstract

A minority of patients presenting to hospital with COVID-19 have bacterial co-infection. Procalcitonin testing may help identify patients for whom antibiotics should be prescribed or withheld. This study describes the use of procalcitonin in English and Welsh hospitals during the first wave of the COVID-19 pandemic. A web-based survey of antimicrobial leads gathered data about the use of procalcitonin testing. Responses were received from 148/151 (98%) eligible hospitals. During the first wave of the COVID-19 pandemic, there was widespread introduction and expansion of PCT use in NHS hospitals. The number of hospitals using PCT in emergency/acute admissions rose from 17 (11%) to 74/146 (50.7%) and use in Intensive Care Units (ICU) increased from 70 (47.6%) to 124/147 (84.4%). This increase happened predominantly in March and April 2020, preceding NICE guidance. Approximately half of hospitals used PCT as a single test to guide decisions to discontinue antibiotics and half used repeated measurements. There was marked variation in the thresholds used for empiric antibiotic cessation and guidance about interpretation of values. Procalcitonin testing has been widely adopted in the NHS during the COVID-19 pandemic in an unevidenced, heterogeneous way and in conflict with relevant NICE guidance. Further research is needed urgently that assesses the impact of this change on antibiotic prescribing and patient safety.

## 1. Introduction

The severe acute respiratory syndrome coronavirus 2 (SARS-CoV-2) has spread as a global pandemic since late 2019. As of 8th February 2021, over 100 million cases and almost 2.5 million deaths related to COVID-19 have been reported globally [1]. Around one-fifth of infections result in severe disease [2] and in the UK, over 400,000 people have been hospitalized with COVID-19 [3].

Although SARS-CoV-2 is a virus, patients hospitalised with COVID-19 frequently receive empiric antibiotics to treat suspected or possible bacterial infection [4]. In the case of influenza, bacterial infection occurs in 11% to 35% of patients, typically involves *S. aureus* or *S. pneumoniae* and is associated with greatly increased mortality [5,6]. Over time, evidence has accrued that bacterial infection during acute COVID-19 is less common, estimated at <10% [7,8]. For COVID-19 patients with the most severe disease, bacterial infection is a diagnosis to be excluded and empiric antibiotics are recommended based on clinical judgement [9,10]. C-reactive protein (CRP) which, in other settings, is widely used as a biomarker of bacterial infection, appears to reflect severity of illness and prognosis in COVID-19, irrespective of the presence of additional bacterial infection [11].

Procalcitonin (PCT) is a polypeptide which has been investigated as a biomarker of bacterial infection since a landmark paper published in 1993 reported the ability of PCT to discriminate bacterial from viral infection [12]. While the performance characteristics of PCT are superior to CRP in distinguishing bacterial from viral infection [13], the role of measuring PCT in antimicrobial stewardship (AMS) is contentious. PCT assays are approved for sepsis and respiratory tract infections by the US Federal Drug Administration, but in the UK, current guidance from the National Institute for Health and Care Excellence (NICE) does not include PCT testing, on the basis of insufficient evidence [14,15]. During the first wave of COVID-19, anecdotal reports suggested some NHS acute hospitals had introduced or expanded use of PCT testing to address concerns about antibiotic overuse in COVID-19 patients. This was despite NICE rapid COVID-19 guidance NG173 advising against routine PCT testing to guide antibiotic prescribing decisions [9].

The Procalcitonin Evaluation of Antibiotic use in COVID-19 Hospitalised patients (PEACH) study [16] has been commissioned and funded by the National Institute of Health Research (NIHR) to evaluate whether the use of PCT testing to guide antibiotic prescribing safely reduced antibiotic use among patients admitted to acute UK NHS hospitals with COVID-19. Here, we describe how acute NHS hospitals used PCT testing to guide antibiotic prescribing during the first wave of the COVID-19 pandemic in England and Wales.

## 2. Results

Responses were received from 148 of 151 (98%) acute hospitals in England and Wales comprising: East of England—16; London—19; Midlands—19; North East and Yorkshire—21; North West—21; South East—17; South West—17; Wales—18. 

**Figure 1:**
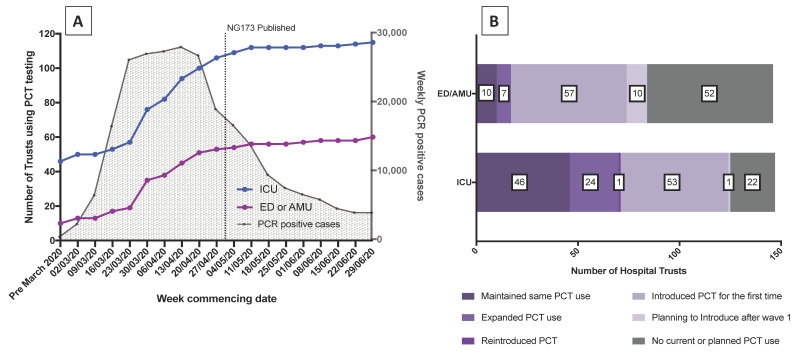
Changed use of PCT testing at acute NHS hospitals in England and Wales. (**A**) Weekly number of hospitals using procalcitonin (PCT) on intensive care unit (ICU) (-•-) and Emergency Department (ED)/Acute Medical Unit (AMU) (-•-). For reference, the time course of the COVID-19 pandemic first wave in England is shown as weekly number of PCR positive cases (-). NG173 = National Institute for Health and Care Excellence (NICE) COVID-19 rapid guideline NG173: [9] Data extracted from Public Health England (PHE) national COVID surveillance reports available at: https://assets.publishing.service.gov.uk/government/uploads/system/uploads/attachment_data/file/916994/COVID19_Weekly_Report_09_September_2020.pdf (accessed on 21 February 2021). (**B**). Breakdown of changes in PCT use on ICU and ED/AMU at 148 hospitals over the course of the pandemic first wave.

Figure 1 shows the change in PCT use during the course of the first wave of the COVID-19 pandemic. Prior to March 2020, PCT was in use for AMS at 70/147 hospitals in ICU (47.6%) and by 17 (11.6%) hospitals in the Emergency Department (ED)/Acute Medical Unit (AMU). Of note, no hospital was using PCT in ED/AMU prior to the COVID-19 pandemic unless it was also in use in ICU.

Many hospitals adopted PCT during the first wave of COVID-19 (Figure 1A); 57/146 (39.0%) of ED/AMUs and 54/147 (36.7%) of ICUs. Most introduced testing in late March and early April as case numbers were increasing rapidly, and well before the publication of NICE rapid guidance NG173 on 1st May 2020. By the end of June 2020, the number of hospitals using PCT on ICU had increased to 124 (84.4% of hospitals) and use in ED/AMU had quadrupled to 74 (50.7% of hospitals), with a further 10 hospitals planning to introduce PCT into ED/AMU after wave 1 (Figure 1B). The discrepancy between the numbers of clinical areas adopting PCT in Graph 1A and 1B is due to missing or inconsistent dates provided by respondents.

In total, 116 respondents completed questions about PCT cut-offs in the ICU, and 78 in non-ICU settings, with most respondents reporting defined cut-offs for stopping or withholding antibacterials in ICU, 107/116 (92.2%), and non-ICU, 68/78 (87.2%) settings (Table 1). The most common cut-off value used on ICU was 0.5 ng/L (54/107 (50.5%) hospitals specifying a level), while outside ICU, 0.25 ng/L was specified at the majority of hospitals (41/68 (60.3%)). Twelve hospitals specified different cut-offs for ICU and non-ICU patients: in 9, the ICU cut-off was higher, and in 3 the non-ICU cut-off was higher. Only a minority of hospitals used PCT results without suggesting a threshold for stopping or withholding antibiotics (9/116 (7.8%) in ICU and 10/78 (12.8%) non-ICU settings.

In total, 114 respondents completed questions about frequency of PCT testing in ICU and 76 in non-ICU settings. Outside ICU, practice around how often to measure PCT was fairly evenly split between taking a single measurement and taking two or serial measurements. On ICU, taking two or more measurements was the norm (Table 1).

Less than half of hospitals, 50/122 (41.0%), using PCT in ICU had it as part of a biochemistry order set and about a third had PCT as part of a biochemistry order set outside ICU (33/107 (30.8%)). Similarly, around a half (55/114 (48.2%)) of hospitals had a guideline for PCT use in AMS.

### Perceptions of the Value of PCT and Future Plans

**Figure 2:**
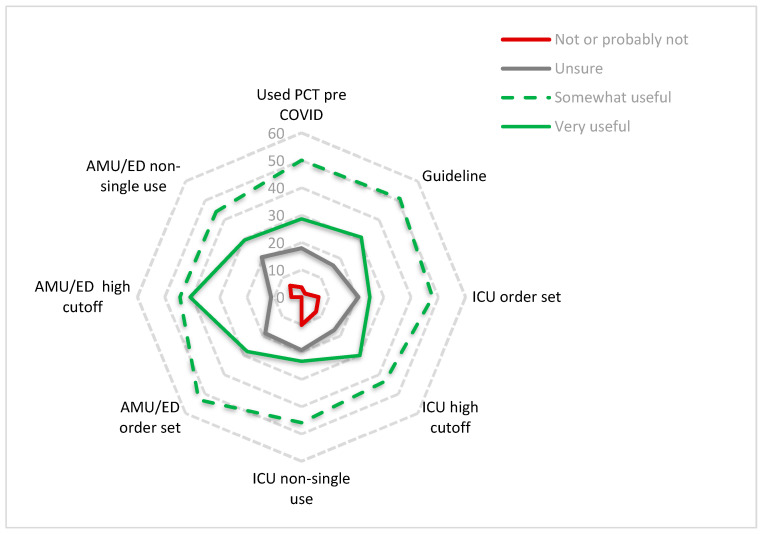
Perceived usefulness of procalcitonin (PCT) in relation to how it was used in practice.

One hundred and fourteen respondents completed questions about perceived value of PCT. The majority of respondents reported that they thought PCT had a positive effect on controlling antibiotic overuse in COVID-19 patients with 78/114 (68.4%) responding with either “Yes somewhat” or “Yes very much”. Twelve out of 114 (10.5%) gave a negative response and thought PCT was “Probably not” or “Not at all” helpful in controlling antibiotic use and 24/114 (21.1%) were unsure. We explored the relationship between perceived value of PCT testing and how the test was introduced (Figure 2). More respondents felt the test was very useful if used with a cut-off of 0.5 ng/L and as part of an order set, particularly in the ED/AMU setting.

The polar plot shows the percentage of respondents who felt PCT testing had been either very useful, somewhat useful, were not sure or not/probably not useful according to whether PCT had been used at their trust pre-COVID, within a guideline, as part of an order set, with a high cut-off (0.5 ng/L) and as non-single use (two or serial measurements) in the ICU or AMU/ED setting.

The majority of ICUs (104/111 (93.7%) that reported PCT use during the first COVID-19 wave plan to continue to use PCT for AMS post COVID. A smaller proportion using PCT in ED and AMU report their intention to continue using PCT in those areas post COVID-19 for AMS; 45/75 (60%) and 60/83 (72.3%), respectively.

## 3. Discussion

Using a survey of hospital antimicrobial pharmacists and doctors from acute NHS hospitals in England and Wales, we found rapid and widespread adoption of PCT testing, particularly in ED/AMU patients with COVID-19; 57 (39%) ED/AMUs and 53 (36%) ICUs newly introduced PCT testing to support AMS during the first wave of the COVID-19 pandemic and 7 (5%) ED/AMUs and 24 (16%) ICUs expanded prior use of PCT to include COVID-19 patients. Implementation was heterogeneous both in terms of the cut-offs used to indicate bacterial infection, and the use of single vs. multiple tests.

Early in the COVID-19 pandemic, it was established that a raised CRP, which is widely used as a biomarker of bacterial infection in the UK, reflected severity of illness rather than bacterial superinfection in COVID-19 patients [17]. While the great majority of COVID-19 patients admitted to hospital in the UK during the first wave received antibiotics, a living meta-analysis of data on the incidence of bacterial infection in patients with COVID-19 demonstrated that <1 in 20 COVID-19 patients had ‘bacterial co-infection’ at presentation (4.9% (95% CI 2.6–7.1)), while 1 in 6 developed ‘secondary bacterial infection’ during their hospital stay (16.0% (95% CI 12.4–19.6)) [18]. Around half the hospitals using PCT in ED/AMU patients used a single test approach and half used two or serial testing, implying a dichotomy of views about how best to use PCT to discontinue antibiotics.

Recognising the potential impact of COVID-19 on antibiotic prescribing, NICE published a COVID-19 rapid guideline (NG173) to assist antibiotic prescribing decisions for pneumonia in hospitalised adults, which recommended basing decisions to start antibiotics on clinical judgement, radiographic changes and neutrophil count [9]. While acknowledging that PCT testing could be used, and encouraging centres using PCT to participate in research, the guidance concluded “there is insufficient evidence to recommend routine procalcitonin testing to guide decisions about antibiotics”. Our data indicate that this guidance was produced after most hospitals had already adopted PCT testing, that most of these hospitals plan to continue to use PCT testing to manage patients with COVID-19 and many hospitals plan to continue to use PCT for AMS post COVID-19, despite being contrary to NICE guidance. We are aware of three UK hospitals who have since published details of their use of PCT to guide antibiotic use in COVID-19 [19,20,21]; these were all retrospective cohort studies, suggesting that antibiotics could be safely withheld with a PCT of <0.25 ng/L, but all findings are limited by uncontrolled confounders. Despite a considerable body of research, the role of PCT as a tool to guide antibiotic prescribing decisions was contentious even prior to the COVID-19 pandemic. The most recent systematic review and meta-analysis relevant to respiratory tract infection considered 6708 participants in 26 randomised controlled trials and concluded that use of PCT to guide initiation and duration of antibiotic treatment resulted in lower risk of mortality, lower antibiotic consumption and fewer antibiotic-related side effects [22]. Nevertheless, while current Infectious Diseases Society of America (IDSA) antimicrobial stewardship guidance supports use of PCT for antibiotic discontinuation decisions in critical care patients [23], neither IDSA nor NICE recommend use of PCT to withhold antibiotics in patients with community acquired pneumonia [9,24]. NICE Diagnostic Guideline DG18 on the use of procalcitonin published in October 2015 concluded there to be insufficient evidence to recommend that these tests are used in the NHS primarily because the PCT studies were done in non-UK healthcare settings and the results, therefore, were not generalisable to a UK healthcare setting [14,15]. NICE have since commissioned three UK PCT studies to address the uncertainty of the efficacy of PCT in a UK healthcare setting: ADAPT Sepsis (adult sepsis in the ICU) [25], PRONTO (adult ED sepsis) [26] and BATCH (paediatric sepsis) [27]. The study protocol for PRONTO has been amended in light of the COVID-19 pandemic to accommodate COVID-19 patients.

We were not able to restrict data entry to a single response per site, so where there were multiple responses, we disregarded incomplete entries, and this may have introduced bias into the data collection process. A strength of this study was the high response rate and triangulation of different data sources in acute NHS hospitals. In spite of this, there were some missing data and a small number of inconsistencies that could not be resolved. Further work within the PEACH [16] study will seek evidence for any impact of PCT testing on antibiotic use and patient outcomes. Nevertheless, by gathering data from almost every acute hospital, we have been able to highlight that PCT testing has been adopted very widely and heterogeneously in the NHS. This adoption has not yet been supported by evidence whether, or how, to use PCT safely and effectively in the context of COVID-19 infection. In 2016, the World Health Organisation published its Guidance for Managing Ethical Issues in Infectious Diseases Outbreaks, building on experience in previous Ebola and Coronavirus epidemics [28]. This highlighted the importance of clinical trials to evaluate treatments of emerging infectious diseases and to limit off-license use only to situations where trials were unavailable. The large UK platform COVID-19 treatment trials such as RECOVERY [29] and PRINCIPLE [30] have successfully limited off-label prescribing in COVID-19, while providing safe and effective platforms for evaluation of treatment options. Our results highlight the need for a similar approach to the deployment of diagnostics in COVID-19 and future pandemics.

## 4. Materials and Methods

A web-based survey was developed by the authors [31]. This sought information about use of PCT for AMS purposes during the first wave of COVID-19 in England and Wales. The survey gathered information about: PCT use prior to the pandemic, whether PCT was adopted during the pandemic and if so, in which areas of the hospital (ICU, ED, AMU), PCT cut-offs, the testing algorithm, whether PCT was part of a hospital guideline or biochemistry order set, whether participants thought PCT was useful in efforts to control antibiotic overuse and whether participants plan to use PCT as part of their antibiotic stewardship program after the first COVID-19 wave. We defined the first wave of COVID-19 as 1 March 2021 through 6 July 2021. The survey was piloted by three hospital AMS leads, and refined. The usability and technical functionality of the electronic questionnaire was tested by the study team before being distributed through UK antimicrobial pharmacist networks by email, Whatsapp and the UK Clinical Pharmacy Network notice board in December 2020 [32]. The survey was voluntary but reminders were sent weekly for three weeks. Outstanding responses were chased personally by members of the study team in January 2021 and the survey closed to responses on 25 January 2021. When more than one survey was submitted from a hospital, the survey containing the greater number of completed fields was included. Any discordant answers were checked with the submitting hospital before removing the duplicate survey. In reporting the survey, we have used the CHERRIES checklist for reporting results of Internet E-Surveys [33]. Ethical approval for the PEACH study was provided by NHS Health Research Authority HRA and Health and Care Research Wales reference 21/WM/0052.

## Figures and Tables

**Table 1:** Nature of procalcitonin (PCT) use to support antibiotic prescribing during first wave of COVID-19 pandemic in England and Wales.

	ICU	Non-ICU
PCT cut-off (ng/L)	n = 116	n = 78
0.1	1(1%)	0
0.2	1 (1%)	0
0.25	51 (44%)	41 (53%)
0.5	54 (47%)	27 (35%)
No cut off specified, cut-off varied dependent on clinical context	9 (8%)	10 (13%)
Timing of PCT testing	n = 114	n = 76
Single measurement	14 (12%)	39 (51%)
Two measurements	23 (20%)	21 (28%)
Serial	72 (63%)	9 (12%)
Other (i.e., varied dependent on clinical context)	5 (4%)	7 (9%)
PCT part of biochemistry order set	n = 122	n = 107
Yes	50 (41%)	33 (31%)
Hospital guideline	n = 114	
PCT part of a Hospital guideline for managing COVID-19	55 (48%)	

## Data Availability

Data available on reasonable request from corresponding author.
